# Improved Captures of the Invasive Brown Marmorated Stink Bug, *Halyomorpha halys*, Using a Novel Multimodal Trap

**DOI:** 10.3390/insects13060527

**Published:** 2022-06-07

**Authors:** Gabriele Rondoni, Elena Chierici, Elisa Marchetti, Stefano Nasi, Roberto Ferrari, Eric Conti

**Affiliations:** 1 Department of Agricultural, Food and Environmental Sciences, University of Perugia, 06121 Perugia, Italy; elenachierici9@gmail.com (E.C.); eric.conti@unipg.it (E.C.); 2 AGRI 2000 Net S.r.l., Castel Maggiore, 40013 Bologna, Italy; marchetti@agri2000.it; 3 MO-EL S.p.A., Montecchio Emilia, 42027 Reggio Emilia, Italy; s.nasi@mo-el.com; 4 Centro Agricoltura Ambiente “Giorgio Nicoli” S.r.l., Crevalcore, 40014 Bologna, Italy; rferrari@caa.it

**Keywords:** attract-and-kill, Hemiptera, integrated pest management, invasive species, LED light, monitoring, Pentatomidae, pest surveillance, pheromone, ultraviolet (UV)

## Abstract

**Simple Summary:**

Pest monitoring using traps is a key component of integrated pest management. For several insects, trapping is achieved using visual or olfactory stimuli. Although the combination of both is supposed to provide higher efficacy, this has often been overlooked in trap design. Through laboratory bioassays and field experiments we evaluated the use of UV-A and visible light in combination with olfactory stimuli to improve trapping of the invasive brown marmorated stink bug, *Halyomorpha halys* (Hemiptera: Pentatomidae). Our results may be useful for the improvement of monitoring strategies for early pest detection. Additionally, the higher efficacy of the multimodal traps would allow their use in attract-and-kill or push–pull strategies within integrated pest management.

**Abstract:**

Capture strategies for the brown marmorated stink bug, *Halyomorpha halys* (Hemiptera: Pentatomidae), are challenging. Here we developed and evaluated a multimodal trap which combines visual and olfactory stimuli. Visual stimuli consisted of LEDs emitting UV-A and visible light. Olfactory stimuli were comprised of the synthetic aggregation pheromone and odours from trapped *H. halys* individuals. Stink bug attraction at different wavelengths was evaluated in laboratory two-choice bioassays, and different prototypes of the trap were tested in 2021 in natural, agricultural, and urban settings. Traps with a combination of UV-A and blue or green visible wavelengths provided higher *H. halys* attraction (up to ~8-fold) compared to traditional sticky or small pyramidal traps. The concurrent presence of synthetic pheromone and LED had a synergistic effect on *H. halys* positive phototaxis. Further development and implementation of the multimodal trap is discussed for prospective use in attract-and-kill or push–pull strategies.

## 1. Introduction

Invasive invertebrates that establish in a new area may cause environmental and economic damage to agriculture and society [1]. From 1970 to 2017 the mean annual cost for damage and management of invasive invertebrates was USD 8.7 billion [2,3]. Management of invasive invertebrates using integrated pest management (IPM) strategies represents an economically effective approach [4,5]. The main strategies for management of invasive invertebrates are based on mechanical, chemical, and biological control tools [6,7,8]. Monitoring is a key element of IPM (Directive 2009/128/EC) and supports farmers in the selection of chemicals and timing of spray applications for managing pests [9]. For example, pheromone-based traps are frequently used and could play a pivotal role in detecting invasive insects as soon as they arrive [10]. 

The brown marmorated stink bug *Halyomorpha halys* Stål (Hemiptera: Pentatomidae) is native to Asia and is now widespread in each continent of the Northern Hemisphere [11]. Its host range includes more than 275 plant species [12], and, in the areas where it has established, it is causing significant agricultural losses [13,14,15]. The first detection in Italy occurred in 2012 in Emilia-Romagna (northern Italy) with more than 50% damage in early maturing pear cultivars being reported [16]. Additionally, during late summer and fall seasons, *H. halys* adults seek shelter within human-made structures to overwinter, thereby being a nuisance to citizens [13,14].

Assessing the density of *H. halys* populations is crucial for anticipating further invasion, allowing growers to determine the best control tools and to ensure timely chemical applications [17]. In recent years, insecticide use has greatly risen for stink bug management [18]. As a consequence, invasion of *H. halys* has resulted in the disruption of IPM programmes for several important crops, with possible non-target effects on beneficial insects, e.g., predators and egg parasitoids notoriously sensitive to lethal and sublethal insecticide doses [19,20,21]. Before becoming invasive, little information was available on the best monitoring tools to be used for *H. halys* [22]. Sweep netting and visual counts were usually employed [23] but often with uncertain results because of stink bugs’ strong dispersal ability [24]. The identification of the two-component aggregation pheromone of *H. halys* [25] and the demonstrated synergistic effect of the aggregation pheromone of another Asian stink bug, *Plautia stali* Scott [26], has provided important insight for pest monitoring. Various studies have assessed the effectiveness of different pheromone-based trap designs, the combination of the pheromones to be employed, and the optimal trap placement [19,27,28]. Noteworthy, is that individuals tend to remain in the proximity of the pheromone trap instead of entering inside it [29]. To overcome this issue, traps combining different stimuli have been developed for monitoring purposes, and, ideally for *H. halys* direct control, e.g., in attract-and-kill or push-pull strategies [30,31]. Vibrational-based signals play an important role in *H. halys* mating behaviour [32], and the addition of such cues to pheromone traps was proven to increase trap efficacy [33]. Rotating live traps captured, on average, 7-fold more *H. halys* adults compared to classic sticky panels [34]. In addition to diurnal feeding activity, stink bugs exhibit intensive nocturnal movement [35]. Hence, light traps, e.g., traps equipped with black light lamps, could be useful tools to investigate population density and seasonal phenology of *H. halys* [35,36]. Black light refers to type-A ultraviolet (UV-A) light with a peak at or near 365 nm and a well-established insect-attracting aptitude [37]. Therefore, to further explore trapping methods for *H. halys*, we tested traps with the combined effect of different stimuli, i.e., LED lights, synthetic pheromone, and natural odours emitted by trapped insects. Captures of insects other than *H. halys* were also recorded to possibly evaluate the effect of such traps on non-targets. Multimodal traps were assessed in agricultural, urban, and natural landscapes of northern and central Italy where *H. halys* is responsible for serious economic losses to crops and citizen nuisance.

## 2. Materials and Methods

### 2.1. Design of the Multimodal Trap

**Figure 1:**
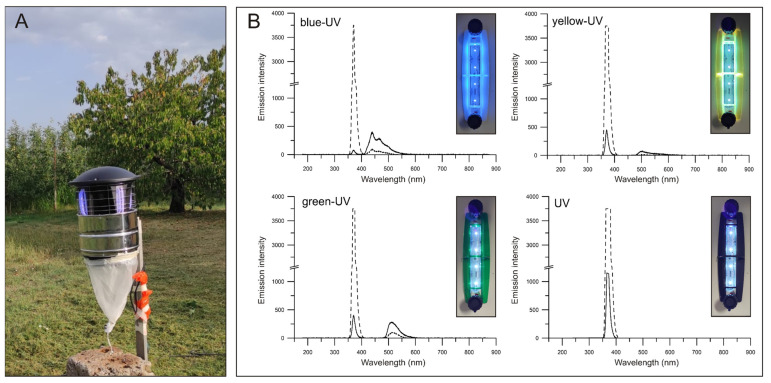
Overview of a multimodal electric trap used in field experiments (**A**). Emission intensity of single lamps emitting blue-UV, green-UV, yellow-UV, or UV only wavelengths were evaluated in laboratory conditions and are reported in arbitrary scale (**B**). For each combination of lamp and plastic grid, the emitted radiation flux was measured at close contact (continuous line) or at 2 cm distance (dashed line).

A trap prototype was developed by MO-EL S.p.A. (Reggio Emilia, Italy) based on their model “TurbiLED” (mod. 806, more details at https://www.mo-el.it/, accessed on 4 May 2022). The trap (Figure 1A and Figure S1) has a cylindrical shape (36 cm diameter, 84 cm height), and the main body is made of steel and black plastic and has four equidistantly spaced openings of approx. 400 cm^2^ each on the side. Between each opening, a digital UV-A LED lamp (10.5 cm length) is vertically fixed on the plastic body of the trap. Basically, each lamp consisted of four LEDs (peak emission at 365 nm) equidistantly positioned every 2.6 cm in a digital strip and is partially covered by a removable and interchangeable plastic grid (14 × 4.2 × 2.1 cm) made of translucid coloured polycarbonate. LED lamps are powered by an electronic converter for LEDs (220 V, 350 mA, Vossloh Schwabe, Sarsina, Italy). When a portion of the UV-A flux intercepts the plastic part of the grid it undergoes a wavelength change, i.e., from 360–380 nm to 380–780 nm, which corresponds to visible light (details in patent EP3909426A1). A second portion of the radiation emitted by LEDs does not intercept the plastic part of the grid and totally consists in UV-A radiation. A motorised fan (details in US20030131525A1) is horizontally placed inside the trap at a central position. By rotating, the fan generates an airstream that sucks the insects surrounding the LED lamp and eventually conveys them inside a polyamide funnel bag placed behind the trap. The trap is completed by a metal grill (approx. 2–4 × 8 cm rectangular holes) externally fixed to prevent the entrance of birds or small mammals into the openings (visible in Figure 1A and Figure S1).

For our experiments, differently coloured plastic grids were evaluated (provided by MO-EL S.p.A.), allowing emission of wavelengths with a dominance of blue (hereafter “blue-UV”), green (“green-UV”), or yellow (“yellow-UV”). A black grid with no reflectance was also tested for emission of UV-only wavelengths. To increase stink bug captures, a dispenser of the aggregation pheromone of *H. halys* (Pherocon^®^, Trécé Inc., Adair, OK, USA) was positioned on the uppermost extremity of the trap. Dispensers comprised both the two-component *H. halys* aggregation pheromone and the synergistic MDT [26]. The novelty of the trap relies on its multimodal characteristics; including, the ability to collect stink bugs through visual stimuli (positive phototaxis toward the combination of UV-A and visible wavelengths) and olfactory stimuli (presence of the aggregation pheromone and odours from trapped individuals which are aerially distributed in the environment as a consequence of the effect of the fan rotation).

### 2.2. Insect Rearing for Laboratory Bioassays

Adults of *H. halys* were collected in northern and central Italy from overwintering sites (January-February 2021) or from agricultural and natural ecosystems (spring 2021) and served to establish a laboratory colony. Clear plastic food containers (30 × 20 × 15 cm) with 5-cm round holes covered by a mesh were used to rear adults in a laboratory under environmentally-controlled conditions (25 ± 1 °C; 16:8 h light/dark). A diet of apples, carrots, green beans, hazelnuts, and sunflower seeds was supplied. Diet was replaced twice a week. *Vicia faba* L. potted plants were placed inside the cages both as a supplementary food source and an oviposition substrate [38]. Eggs were collected daily and transferred to new cages for rearing of the offspring. Water was provided daily on wetted cotton in a Petri dish. Males and females in reproductive stages were used for experiments.

### 2.3. Measurement of the Radiation Flux and Laboratory Evaluation of H. halys Phototaxis

The radiation flux emitted by the different combinations of UV lamp and plastic grids was measured in the laboratory by positioning the sensor of a digital spectrophotometer (mod. USB2000, Ocean Optics, Ostfildern, Germany) at close contact or at 2 cm distance from the plastic grid covering the LED lamp. Emission intensity was measured up to 3750 photon count.

For the evaluation of *H. halys* phototactic behaviour, bioassays consisted of two-choice tests and were performed in a dark room maintained at a constant temperature of 25 ± 1 °C. Groups of 5 adult females or males were isolated in 50 mL Falcon tubes closed with a small net to allow aeration. Three hazelnut fruits were supplied as food, and insects were kept in the bioassay room for 1 h before being tested. One stink bug at time was assayed. 

Bioassays were conducted on the surface of a black table (100 × 80 cm). Two visual stimuli were both provided at one of the longest sides of the table. The two stimuli were positioned at 60 cm distance and were separated by a black cardboard panel (length: 40 cm, height: 60 cm) to minimise interference between the two stimuli.

Treatments consisted of one UV lamp with four LEDs partially covered by a plastic grid (as described in Section 2.1) and vertically affixed on an electric box (10 × 16 × 7 cm), used as support. For all bioassays, one choice consisted of a blue-UV light. The other choice consisted in control stimulus (light off), or in UV, yellow-UV, or green-UV light. 

A line was drawn on the table at 10 cm from each stimulus source. Each stink bug was released from the opposite side of the stimuli and allowed to freely move on the table surface and eventually choose between one of the two stimuli. A choice was considered when the insect passed the line and remained in the area close of the stimulus for at least 10 seconds. After that, the bioassay was ended. Each insect was observed for a maximum of 5 min. Stink bugs were assayed once and not re-used. The number of replications ranged from 53 to 81 for females and from 56 to 81 for males.

### 2.4. Field Experiments

Five experiments were conducted at locations in northern and central Italy with different habitat types (details of the experiments are presented in Table 1). Various multimodal trap settings were evaluated for attraction and trapping of *H. halys* and/or other insect groups. All multimodal and control traps, except for one in experiment 5, were provided with the aggregation pheromone lure (as in Section 2.1). Samplings were conducted daily at different times (details in Table 1). When one trap with differently coloured lights was used (experiments 1 and 2), the trap was rotated 90° after each sampling. In the other experiments, the position of all traps was changed after each sampling (experiments 3 and 5), or after two consecutive samplings (experiment 4).

Experiments 1 and 2 were conducted in a conservation area in northern Italy near Montechiarugolo, Reggio Emilia (coordinates: 44.69330, 10.42654) between July and August 2021. A multimodal trap with 4 lamps emitting blue-UV, green-UV, yellow-UV, or UV-only wavelengths (experiment 1) and a trap with 2 lamps emitting blue-UV or green-UV (experiment 2) light were assessed for *H. halys* attraction. For experiment 1, two samplings were conducted over two consecutive days. For experiment 2, one sampling was conducted and repeated over four consecutive days. In these experiments the arrestment of *H. halys*, i.e., the number of insects that were present in each sector of the trap at each observation, was evaluated.

Experiments 3 and 4 were conducted in a fruit orchard in central Italy near Ponte Pattoli, Perugia (coordinates: 43.17663, 12.44938) between August and September 2021. The difference in daily capture among the multimodal trap and two different commercial traps as control was evaluated. The multimodal traps were equipped with 4 lamps emitting green-UV or blue-UV light. In experiment 3, a total of 3 samplings were conducted; only one trap for each type was positioned and only data on *H. halys* were collected. In experiment 4 (6 replicates in total), two traps per type were used and both *H. halys* and other insect groups were assessed. Controls were a pyramidal trap (Rescue, Sterling International Inc., Sokane, WA, USA) in experiment 3 and sticky traps (Certis, Milano, Italy) in experiment 4.

Experiment 5 was conducted in an urban park in northern Italy near Crevalcore, Bologna (coordinates: 44.71658, 11.14878) between August and September 2021. Three multimodal traps with different equipment were compared. These were: a multimodal trap with 4 blue-UV lamps and the pheromone lure, a trap with the pheromone lure but with the LED lamps turned off, a trap with the LED lamps turned on but without pheromone lure. Eleven samplings were conducted.

### 2.5. Statistical Analysis

For laboratory bioassays, generalised linear models (GLMs, logit link, Binomial error distribution) were fitted to test the first choice, i.e., the area close to the stimulus the stink bug entered first [39]. For all field experiments, treatment replications were split across different days. In addition, for experiment 4, true replications (2) were also conducted. Linear mixed-effects models (LMMs) were used to analyse the fixed effect of experimental treatments and the random effect of samplings on insect abundance (two factors complete-block design, [40]). The relevance of the random term was evaluated with a likelihood ratio test [41]. In those cases where its presence was not justified, only the fixed term was retained in a linear model (LM) [41]. Abundance data were box-cox transformed before analysis. Multiple comparisons were eventually conducted, adopting the Sidak correction. Analyses were conducted under R statistical environment, packages “MASS”, “nlme” and “emmeans” [42].

## 3. Results

### 3.1. Measurement of the Radiation Flux and Laboratory Evaluation of H. halys Phototaxis

The measurement of the radiation flux for blue-UV, green-UV, yellow-UV, or UV light revealed that the higher portion of the emission was represented by undisturbed UV-A wavelengths, and only a small fraction constituted visible light (Figure 1B). 

**Figure 2:**
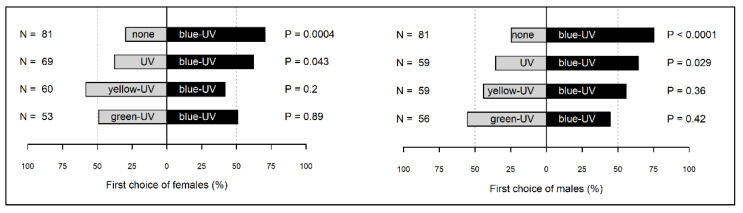
First choice (%) of *Halyomorpha halys* females and males in a two-choice experimental setup. One choice (black bar) consisted of blue-UV lamp. The other choice (grey bar) consisted of the control stimulus (no light and no grid), or in a single lamp emitting UV, yellow-UV or green-UV light. For each comparison, *H. halys* preference was analysed by means of binomial GLM.

In laboratory conditions, female *H. halys* were highly attracted to visual stimuli from blue-UV light compared to control (no light) (Binomial GLM, *p* = 0.0004, Figure 2) or to UV-only light (*p* = 0.043). No differences were detected between blue-UV and yellow-UV, or green-UV (*p* > 0.05 for both comparisons) light. Male *H. halys* where highly attracted towards visual stimuli from blue-UV compared to control (*p* < 0.0001) or to UV-only (*p* = 0.029). No differences were detected between blue-UV and yellow-UV or green-UV (*p* > 0.05 for both comparisons). 

### 3.2. Field Experiments

Experiment 1: When the blue-UV, green-UV, yellow-UV, and UV light were compared in the same trap, the presence of the stink bugs (arrestment on the trap sector corresponding to a different light type) was higher in relation to green-UV light compared to all other treatments (results of LMM followed by multiple comparison procedure are reported in Table 2). Notably, the stink bug presence changed across the different observation periods (ΔAIC = 9.33, *p* = 0.0008). Experiment 2: When only blue-UV and green-UV light were compared in the same trap, the presence of the stink bugs was similar across treatments (ΔAIC = 1.93, *p* = 0.80) and sampling days (ΔAIC = 1.50, *p* = 0.48) (Table 2). Experiment 3: When blue-UV and green-UV light were compared in different traps, *H. halys* captures were similar across sampling days (ΔAIC = 2.00, *p* = 1.00) but higher for the blue and green traps compared to control trap (results of LM followed by multiple comparison procedure are reported in Table 2).

Experiment 4: Captures of *H. halys* were higher for both blue-UV and green-UV traps compared to sticky traps (Table 3). Similar patterns were detected for other Pentatomidae, Hemiptera, Lepidoptera, Coleoptera, Diptera, and Hymenoptera. Random term for sampling dates was never justified except for Coleoptera (ΔAIC = 2.60, *p* = 0.030).

Experiment 5: When captures were compared among a trap with blue-UV light and pheromone, a trap with pheromone only, and a trap with blue-UV light only, higher *H. halys* captures were detected for the complete multimodal trap compared to the other treatments (results of LM followed by multiple comparison procedure are reported in Table 4). Capture of noctuid moths (Lepidoptera: Noctuidae) changed across sampling days (ΔAIC = 2.28, *p* = 0.038) and was higher for the two treatments with light compared to the treatment with pheromone only. Captures of ladybird beetles (Coleoptera: Coccinellidae) were stable across sampling days (ΔAIC = 2.0, *p* = 1.00) and did not differ across treatments (ΔAIC = 1.30, *p* = 0.090). Captures of wasp-waisted wasps (Hymenoptera: Apocrita) were different across sampling days (ΔAIC = 0.97, *p* = 0.085) and were higher for the two treatments with lights compared to the treatments with only pheromone.

## 4. Discussion

In agricultural ecosystems, early detection of a herbivorous insect population is crucial for effective pest management [43,44]. Hence, the improvement of insect trapping is a priority [34,45]. Our results revealed that, in laboratory and field conditions, *H. halys* males and females were attracted toward LED lights emitting both UV-A and visible wavelengths. In addition, the combination of such lights with odour stimuli resulted in higher captures compared to pheromone-based traps or light traps alone.

It is already known that *H. halys*, as well as herbivorous insects in general, including other stink bugs, are attracted by different light wavelengths [46]. In a free-flying experiment testing LEDs with emission of different wavelengths, both male and female *Nezara viridula* (L.) (Hemiptera: Pentatomidae) exhibited stronger attraction toward UV compared to visible wavelengths, and secondarily toward blue and green compared to orange light [47]. In dual-choice laboratory bioassays, *H. halys* showed positive phototaxis toward fluorescent black light (with a UV dominant output) or blue light (with a dominant output in the visible spectrum and no emission in the UV spectrum) [48]. Attraction to UV-dominant lamps was only marginally higher compared to the blue lamp. Interestingly, our results support laboratory results in [48], as it seems that the combination of UV and visible blue light provides higher attraction compared to UV alone. In another experiment, white, yellow, red, orange, and green source lights were attractive to *H. halys* [49]. Similarly, in our laboratory experiments, it seems that *H. halys* can detect and are attracted by either blue, green, or yellow light. Most insects have three types of pigments that ensure UV-blue-green trichromacy [50]. A recent genome analysis revealed the presence in *H. halys* of a singleton UV-opsin homolog and duplicates of long-wavelength opsin homologs but lacked the presence of blue-sensitive opsin ortholog [51]. In other insect orders, the expansion of long-wavelength sensitive opsins can restore trichromacy, but whether this is also likely for *H. halys* remains to be investigated [52,53]. It is possible that gene duplication may have occurred due to the need for wider colour vision. Apparently, from our field experiment 1, it could be expected that a multimodal trap with only green-UV light would capture more *H. halys* compared to other wavelengths, including blue-UV. However, subsequent field experiments revealed that green-UV or blue-UV multimodal traps captured similar numbers of *H. halys*. A speculative explanation is that because *H. halys* has a marked preference for immature and mature fruits, this species has evolved photoreceptors to better discriminate green and longer (yellow or red) wavelengths, and that such preference is only evident during the photophase (i.e., when experiment 1 was conducted). However, further experiments should aim at clarifying such findings.

Although light and pheromone alone were similarly attractive, the simultaneous presence of both stimuli synergistically improved the attractiveness. This result is consistent with a previous investigation where the combination of fluorescent blue light and aggregation pheromone increased mid-season *H. halys* trapping in mid-Atlantic sites of the US [24]. Modification of the trap design consisted of the addition of an electric fan. The light lamp attracted insects to the trap, but they tended to remain close to the light source [49] and were reluctant to enter inside the funnel bag [Authors personal observation]. The presence of the fan creates a suction vortex that possibly facilitated the movement of the stink bugs to the funnel net cage (similarly to [45]). The fan could help the dispersal of synthetic and natural aggregation pheromone. In Maryland, traps with live *H. halys* individuals successfully attracted conspecifics, possibly because of the production of natural aggregation pheromone from trapped individuals [29]. Similarly, in Italy, a prototype of a live trap ensured higher *H. halys* captures compared to a control trap [34]. In a soybean field in Miryang, Korea, the presence of a solar fan in pheromone baited traps improved captures of the stink bugs *Riptortus pedestris*, *H. halys*, and *Piezodorus hybneri* [54]. The addition of a blue LED lamp (no UV emission) to the traps further increased *H. halys* trapping [45]. In our experiment we left trapped adults for only one day before removal. Hence, further investigations would help clarify whether leaving trapped adults for a prolonged time would increase trap efficacy, or otherwise, decrease it due to possible emission by trapped individuals of defensive compounds (e.g., aldehydes, with demonstrated repellent activities on conspecifics) [55]. Another aspect is that insect populations of diverse geographical origins may exhibit different behaviour (e.g., the stem borers *Sesamia nonagroides* Lefèbvre [56,57] and *Chilo partellus* (Swinhoe) [58]). Hence, whether different populations of *H. halys* similarly respond to UV-A and visible wavelengths must be verified.

Non-target insect trapping is a side effect of traditional light traps [59,60]. In our multimodal traps we noticed generally low captures of Coccinellidae. This group was indeed abundant in the urban and agricultural systems under evaluation and included native and exotic species which provide effective control over herbivorous insect pests [61,62]. Previous studies investigated the phototactic behaviour of ladybirds to different wavelengths and have reported light response variability among different species and insect physiological status. In laboratory conditions, *Coccinella septempunctata* L. (Coleoptera: Coccinellidae) exhibited positive phototactic behaviour toward UV and red lights and much lower response to green or blue lights [63]. Notwithstanding, captures by blacklight UV traps in field trials were sporadic for several ladybird species, including *C. septempunctata* [64]. Conversely, *Harmonia axyridis* (Pallas) (Coleoptera: Coccinellidae) was strongly attracted by UV and blue wavelengths [64,65]. Despite this species providing effective pest suppression [66], it is considered invasive in several parts of the world and puts native predators at risk due to competition for resources or intraguild predation [67,68,69]. Hence, an interesting opportunity would be the evaluation and definition of proper wavelengths that might reduce *H. axyridis* populations in those areas where this species has become a concern for threating insect biodiversity [70]. Notably, even if the multimodal trap captured some Hymenopterans, we counted very few honeybees and bumblebees. In fact, blue wavelengths are non-preferential for these beneficial insects [71,72]. Conversely, several Lepidoptera were found inside blue-UV and green-UV traps, and it is known that UV and blue wavelengths, are particularly attractive for them [73]. Because light attraction seems to be positively correlated with the size of the moth [74], the prototype of the multimodal traps could be improved. For instance, it is expected that the adoption of an external metal grill with smaller openings would likely prevent trapping of large Lepidoptera. The large number of Diptera captured in our multimodal traps can be explained by the peak sensitivity of their compound eye retinula cells to the UV, green, and blue wavelengths [75].

Concerning IPM, the multimodal trap that we evaluated could be used not only for pest monitoring, but also for directly controlling *H. halys*, e.g., through mass trapping [30]. Additionally, such traps could also be adopted in combination with repellent molecules (e.g., terpenes) in a push–pull strategy. Such IPM approaches would likely enhance *H. halys* control, also because it is expected to have low interference with complementary control practices, such as biocontrol with native and exotic parasitoids [76,77,78,79,80,81]. Concerning practicality of field use, our prototype is based on the TurbiLED trap which has a IPX4 waterproof level. That means the trap can be used externally and risk of water infiltration, e.g., by heavy rain, can be disregarded. A new prototype powered by a 12 V battery recharged by a solar panel is currently under development.

## 5. Conclusions

We have evaluated a multimodal trap that combines visual and olfactory stimuli and greatly enhanced *H. halys* captures. Other research would be needed to better understand the *H. halys* phototaxis behaviour in different environmental conditions. For example, the response at different light intensities [49], evaluation time (which could be related to diurnal and nocturnal circadian rhythm [60]), or physiological status [64]. Furthermore, phototaxis response of non-target insects should be properly evaluated in order to possibly reduce undesired biodiversity losses [50].

## Figures and Tables

**Table 1:** Overview of the different field experiments (Exp).

Habitat Type	Exp	Date (2021); Time of Samplings [Number of Samplings]	Type of Trap	Type of Stimuli Evaluated	Phero-Mone	Number of Traps Used	Type of Data Collected
Conservation area	1	27–28 July; 8:00–9:00 and 17:00–18:00 [4]	Multimodal trap with 4 lamps, each of a different colour.	Blue-UV, green-UV, yellow-UV, UV	Yes	1	arrestment of *H. halys* on trap surface during photophase
	2	29 July–01 August; 8:00–9:00 [4]	Multimodal trap with 2 lamps, each of a different colour.	Blue-UV, green-UV	Yes	1	
Fruit orchard	3	18–19 August, 23–24 August, 25–26 August; 8:00–9:00 [3]	Multimodal trap with 4 lamps of the same colour. A pyramidal trap (Rescue) was used as control.	Blue-UV, green-UV	Yes	1 of each type	capture of *H. halys* in 24 h
	4	26–28 August, 02–04 September, 13–15 September; 8:00–9:00 [6]	Multimodal trap with 4 lamps of the same colour. Clear sticky traps were used as controls.	Blue-UV, green-UV	Yes	2 of each type	capture of *H. halys* and other insect groups in 24 h
Urban park	5	31 August–11 September; 17:00–18:00 [11]	Multimodal trap with 4 lamps of the same colour.	Blue-UV + pheromone, blue-UV only, pheromone only	Yes/no	1 of each type	capture of *H. halys* and other insect groups in 24 h

**Table 2:** Arrestment/presence (mean ± SE) of *H. halys* in the different sectors of a single multimodal trap (Exp. 1 and 2) or total *H. halys* (mean ± SE) captured in multimodal or control traps (Exp. 3). For each experiment, means followed by different letters are significantly different according to linear mixed-effects model followed by multiple comparisons procedure (significance level α = 0.05).

Exp	Treatment	*H. halys*
1	Blue-UV	4.00 ± 2.04 ^b^
	Green-UV	19.00 ± 7.12 ^a^
	Yellow-UV	2.25 ± 1.03 ^b^
	UV	3.00 ± 1.78 ^b^
2	Blue-UV	14.00 ± 3.54
	Green-UV	16.50 ± 5.92
3	Blue-UV	34.33 ± 5.04 ^a^
	Green-UV	33.00 ± 5.13 ^a^
	Control (pyramidal trap)	4.00 ± 2.08 ^b^

**Table 3:** Captures (mean ± SE) of *H. halys* and other main insect groups in multimodal and control traps (Exp. 4). Means in the same column followed by different letters are significantly different according to linear model or linear mixed-effects models followed by multiple comparisons procedure (significance level α = 0.05).

Exp	Treatment	Hemiptera (*H. halys*)	Hemiptera (Other Pentatomidae)	Hemiptera (Other)	Lepidoptera	Coleoptera	Diptera	Hymenoptera
4	Blue-UV	11.33 ± 2.93 ^a^	6.08 ± 1.33 ^a^	65.92 ± 21.52 ^a^	269.25 ± 38.47 ^a^	27.92 ± 5.74 ^a^	799.58 ± 121.41 ^a^	26.42 ± 7.42 ^a^
	Green-UV	12.67 ± 3.07 ^a^	6.33 ± 1.14 ^a^	72.5 ± 26.42 ^a^	210.75 ± 32.77 ^a^	21.75 ± 4.78 ^a^	722.42 ± 162.29 ^a^	22.33 ± 4.53 ^a^
	Control (sticky trap)	2.83 ± 0.73 ^b^	0.92 ± 0.67 ^b^	0.17 ± 0.17 ^b^	1.33 ± 0.43 ^b^	0.00 ± 0.00 ^b^	10.25 ± 6.63 ^b^	3.42 ± 1.44 ^b^

**Table 4:** Captures (mean ± SE) of *H. halys* and other main groups in a standard or simplified multimodal trap (Exp. 5). Means in the same column followed by different letters are significantly different according to linear model or linear mixed-effects models followed by multiple comparisons procedure (significance level α = 0.05).

Exp	Treatment	*H. halys*	Lepidoptera: Noctuidae	Coleoptera: Coccinellidae	Hymenoptera: Apocrita
5	Blue-UV + pheromone	28.00 ± 5.09 ^a^	42.45 ± 5.04 ^a^	1.45 ± 0.47	19.00 ± 6.94 ^a^
	Blue-UV only	9.00 ± 1.60 ^b^	45.45 ± 10.65 ^a^	1.27 ± 0.60	23.55 ± 9.78 ^a^
	Pheromone only	11.55 ± 1.36 ^b^	5.09 ± 0.81 ^b^	0.18 ± 0.12	2.18 ± 0.48 ^b^

## Data Availability

The data presented in this study are available upon request from the corresponding author.

## Supplementary Materials

The following supporting information can be downloaded at: https://www.mdpi.com/article/10.3390/insects13060527/s1, Figure S1: Diagram of the multimodal trap.
