# Supplementary material for: Improved Captures of the Invasive Brown Marmorated Stink Bug, Halyomorpha halys, Using a Novel Multimodal Trap
Source: Insects. 2022 Jun 7;13(6):527. doi: 10.3390/insects13060527 (PMC9224681; doi:10.3390/insects13060527)
Supplement: Supplementary file 1 [file insects-13-00527-s001.zip › insects-1739361-supplementary.pdf]

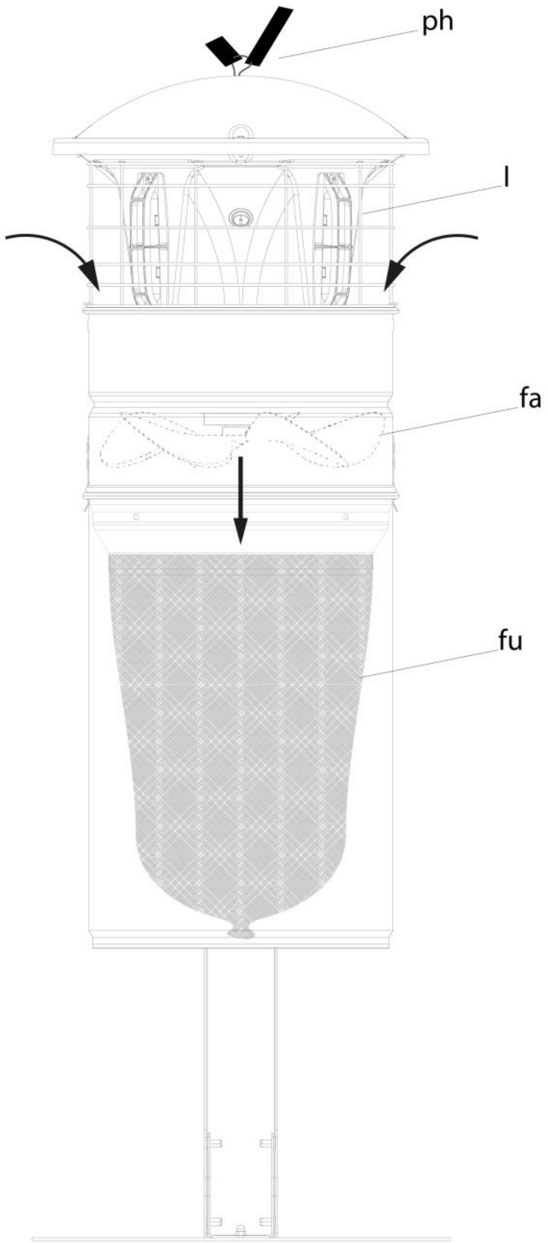

Figure S1. Diagram of the multimodal trap. "ph"= pheromonal lure; "l" = LED light covered by a green-emitting light plastic; "fa"= electric fan; "fu"= collection funnel
